# Location matters: altered interhemispheric homotopic connectivity in post-stroke dyskinesia

**DOI:** 10.3389/fneur.2024.1308058

**Published:** 2024-04-30

**Authors:** Changjiang Zhao, Can Zhang, Li Zhu, Long Chen, Xiong Xiong, Junlong Pan, Jiangjin Chen, Lei Gao, Chengxin Yu, Haibo Xu

**Affiliations:** ^1^Department of Radiology, Zhongnan Hospital of Wuhan University, Wuhan, Hubei, China; ^2^Department of Radiology, The First College of Clinical Medical Science of China Three Gorges University, Yichang, Hubei, China

**Keywords:** stroke, motor function, functional connectivity, structural connectivity, MRI

## Abstract

**Background:**

Motor impairment is the most prevalent consequence following a stroke. Interhemispheric homotopic connectivity, which varies regionally and hierarchically along the axis of the somatomotor-association cortex, plays a critical role in sustaining normal motor functions. However, the impact of strokes occurring in various locations on homotopic connectivity is not fully understood. This study aimed to explore how motor deficits resulting from acute strokes in different locations influence homotopic connectivity.

**Methods:**

Eighty-four acute ischemic stroke patients with dyskinesia were recruited and divided into four demographically-matched subgroups based on stroke locations: Group 1 (G1; frontoparietal, *n* = 15), Group 2 (G2; radiation coronal, *n* = 16), Group 3 (G3; basal ganglia, *n* = 30), and Group 4 (G4; brain stem, *n* = 23). An additional 37 demographically-matched healthy controls were also recruited in the study. Multimodal MRI data, motor function assessments, and cognitive tests were gathered for analysis. Interhemispheric homotopic functional and structural connectivity were measured using resting-state functional MRI and diffusion tensor imaging, respectively. These measurements were then correlated with motor function scores to investigate the relationships.

**Results:**

Voxel-mirrored homotopic connectivity (VMHC) analysis showed that strokes in the frontoparietal and basal ganglia regions led to diminished homotopic connectivity in the somatosensory/motor cortex. In contrast, strokes in the radiation coronal and brainstem regions affected subcortical motor circuits. Structural homotopic connectivity analysis using diffusion tensor imaging showed that frontoparietal and basal ganglia strokes predominantly affected association fibers, while radiation coronal and brainstem strokes caused widespread disruption in the integrity of both cortical-cortical and cortical-subcortical white matter fibers. Correlation analyses demonstrated significant associations between the Fugl-Meyer Assessment (FMA), Modified Barthel Index (MBI), and National Institutes of Health Stroke Scale (NIHSS) scores with the VMHC in the inferior temporal gyrus for G1 (G1; *r* = 0.838, *p* < 0.001; *r* = 0.793, *p* < 0.001; and *r* = −0.834, *p* < 0.001, respectively). No statistically significant associations were observed in Groups 2, 3, and 4.

**Conclusion:**

Our results suggest that motor deficits following strokes in various regions involve distinct pathways from cortical to subcortical areas. Alterations in lesion topography and regional functional homotopy provide new insights into the understanding of neural underpinnings of post-stroke dyskinesia.

## 1 Introduction

Ischemic stroke constitutes ~80% of all stroke cases (1, 2), and results in various outcomes, ranging from sensory and motor impairments to cognitive and consciousness disorders. Among these consequences, motor dysfunction is the most common sequelae (3). However, the neurobiological underpinnings that explain how ischemic lesions in different brain regions collectively contribute to motor impairments remain largely unclear. Gaining insight into the mechanisms of motor circuit damage caused by strokes in various locations is essential for predicting patient outcomes and devising effective treatments.

Normal motor function relies on a delicate balance between excitation and inhibition in interhemispheric interactions (4–7). Advanced MRI neuroimaging provides a non-invasive approach to measure interhemispheric connectivity. Functionally, interhemispheric connectivity can be measured by blood oxygenation level-dependent (BOLD) (8) signals of resting-state functional MRI (R-fMRI) (9, 10). A popular and well-calibrated methodology is functional homotopy, which can be obtained e.g., by voxel-mirrored homotopic connectivity (VMHC) (11). Structurally, diffusion tensor imaging (DTI) enables the non-invasive examination of interhemispheric anatomical connectivity by tracking the movement of water molecules (12, 13). Together, these techniques offer complementary insights into the structural and functional relationships between hemispheres (14). The application of VMHC to assess interhemispheric connectivity in movement disorders has been extensively documented. For example, motor disorders including chronic subcortical stroke (15), subacute stroke (16), dyskinetic cerebral palsy (17), Parkinson's disease patients with levodopa-induced dyskinesias (18) have all demonstrated generally consistent reductions in VMHC across motor, visual, and motor-control regions. Conversely, in studies, of less common movement disorders like Paroxysmal kinesigenic dyskinesia, increased VMHC has been observed in the basal ganglia-thalamo-cortical circuitry and cerebellum (19). Furthermore, other studies have identified both decreases and increases in VMHC associated with movement disorders (20, 21), suggesting that dyskinesias may result from variations in the coordination of interhemispheric spontaneous activity across different anatomical locations, exhibiting interregional variability in excitation and inhibition. To test this hypothesis, we collected data from a group (*n* = 84) of subacute ischemic stroke patients with motor deficits. We divided these patients into four subgroups based on the location of their stroke: frontal-parietal (G1), subcortical radial corona (G2), basal ganglia (G3), and brainstem (G4). Our goal was to examine alterations in their homotopic connectivity and the relationship of these changes to motor function results.

## 2 Materials and methods

### 2.1 Participants

This study comprised 84 patients with acute ischemic stroke, divided into four demographically matched subgroups based on the stroke locations: Group 1 (G1; frontoparietal, *n* = 15), Group 2 (G2; radiation coronal, *n* = 16), Group 3 (G3; basal ganglia, *n* = 30), and Group 4 (G4; brain stem, *n* = 23). Concurrently, we recruited 37 stroke-free healthy controls (HC), matched to the patient subgroups by underlying diseases and demographics. Recruitment spanned from February 2022 to October 2022. The inclusion criteria for patients were: (1) ages 40–80 years; (2) right-handedness; (3) confirmed diagnosis of acute ischemic stroke with stabilized vital signs; and (4) neurologic physical examination confirming dyskinesia, with and diffusion-weighted imaging (DWI) indicative of a focal stroke lesion. Exclusion criteria for all participants were: (1) cerebrovascular progression or unstable vital signs; (2) other brain abnormalities, clinically significant or unstable diseases, or inability to perform daily activities independently before stroke; (3) use of antiepileptic or antipsychotic medications affecting motor function evaluation; or (4) MRI contraindications. The present study received approval from the Ethics Committee of The First College of Clinical Medical Science of China Three Gorges University. In accordance with the Helsinki Declaration, written informed consent was obtained from each participant and their respective guardian prior to data collection. Table 1 presents comprehensive demographic data and clinical measures.

**Table 1:** Demographic and clinical characteristics.

	**G1 (*n* = 15)**	**G2 (*n* = 16)**	**G3 (*n* = 30)**	**G4 (*n* = 23)**	**HC (*n* = 37)**	***F*/χ^2^**	***p***
Gender (M/F)	10/5	9/7	19/11	16/7	21/16	1.376	0.844^a^
Age (yrs.)	63.92 ± 8.58	65.82 ± 7.24	60.20 ± 8.04	61.14 ± 6.20	61.51 ± 8.95	1.093	0.365^b^
Education (yrs.)	9.85 ± 3.67	9.36 ± 3.91	9.80 ± 3.27	10.07 ± 3.83	10.05 ± 3.54	0.091	0.985^b^
Hypertension	9 (60%)	10 (62.50%)	18 (60%)	15 (65.22%)	24 (64.86%)	0.277	0.991^a^
Smoke	6 (40%)	5 (31.25%)	9 (30%)	9 (39.13%)	13 (35.14%)	1.010	0.908^a^
Drinking	7 (46.67%)	5 (31.25%)	12 (40%)	9 (39.13%)	11 (29.73%)	1.820	0.769^a^
NIHSS	3.15 ± 4.08	4.09 ± 2.95	5.50 ± 5.28	3.86 ± 2.51	0.00 ± 0.00	11.755	< 0.001^b^
FMA	76.23 ± 8.90	61.91 ± 10.33	64.75 ± 8.42	72.43 ± 6.69	100.00 ± 0.00	12.504	< 0.001^b^
MMSE	26.00 ± 4.18	23.45 ± 6.68	24.60 ± 5.57	28.07 ± 2.76	27.03 ± 2.54	2.971	0.024^b^
MBI	75.54 ± 35.82	62.64 ± 30.78	65.30 ± 31.74	63.07 ± 27.81	100.00 ± 0.00	11.110	< 0.001^b^

^a^p-value obtained by a two-tailed Pearson chi-square test.

^b^p-value obtained by a one-way ANOVA. Values are presented as mean ± (SD) or number (%). G1, frontal-parietal; G2, radiation coronal; G3, basal ganglia; and G4, brainstem stroke subgroups, respectively.

M, male; F, female; NHISS, National Institute of Health Stroke Scale; FMA, Fugl-Meyer Assessment; MMSE, Mini-Mental State Examination; MBI, Modified Barthel Index.

### 2.2 Motor and cognitive function assessment

On the day of the fMRI scan, various clinical behavior scores were collected. These included the National Institutes of Health Stroke Scale (NIHSS) (22) to assess neurological deficits, the Mini-Mental State Examination (MMSE) (23) for evaluating global cognitive capacities, the Fugl-Meyer Assessment (FMA) (24) to measure the extent of motor impairment in the upper and lower extremities, and the Modified Barthel Index (MBI) (25) for assessing an individual's ability to perform activities of daily living. Together, these scores provided a comprehensive evaluation of the stroke patients' neurological and functional status.

### 2.3 MRI data acquisition

All MRI data were obtained using a Philips Ingenia 3.0 T MR scanner (Philips Medical Systems, Best, the Netherlands). The scanning protocol included: (1) three-dimensional T1 high-resolution anatomical images [repetition time (TR) = 7.9 ms, echo time (TE) = 3.8 ms, flip angle (FA) = 8°, field of view (FOV) = 250 × 250 × 181 mm^3^, voxel size = 1.0 × 1.0 × 1.0 mm^3^, matrix size = 228 × 227, slice thickness = 1.0 mm]; (2) resting-state fMRI (TR = 2,000 ms, TE = 22 ms, FA = 90°, FOV = 192 × 192 × 144 mm^3^, voxel size = 3 × 3 × 3 mm^3^, matrix size = 64 × 64, slice thickness = 3 mm, no gap, 48 interleaved transversal slices with a total of 240 repetitions); (3) T2-weighted imaging (TR = 3,000 ms, TE = 80 ms, FOV = 240 mm × 240 mm, matrix size = 256 × 256, slice thickness = 5 mm, gap = 1 mm); (4) T2- fluid attenuated inversion recovery (FLAIR; TR = 9,000 ms, TE = 120 ms, FOV = 240 mm × 240 mm, matrix size = 256 × 256, slice thickness = 5 mm, gap = 1 mm); (5) DWI (TR = 1,500 ms, TE = 52 ms, FOV = 240 mm × 240 mm, matrix size = 120 × 109, slice thickness = 5 mm, gap = 1 mm, b values = 0 and 1,000 s/mm^2^); and (6) diffusion tensor imaging (DTI; TR = 3,593 ms, TE = 77 ms, FOV = 224 × 224 × 132 mm^3^, voxel size = 2 × 2 × 2 mm^3^, matrix size = 112 × 109, slice thickness = 2.0 mm, no gap, b values = 0 and 800 s/mm^2^, a total of 66 axial slices acquired in 32 directions). During the resting-state fMRI scan, participants were asked to maintain still, close their eyes, refrain from falling asleep, and avoid engaging in systematic thinking.

### 2.4 Lesion segment

Stroke lesions were manually segmented using ITK-SNAP software (Ver 3.8.0; https://www.itksnap.org/). Routine MRI sequences including DWI, conventional T2- and T1-weighted images were acquired on the 1st day following patient admission. On the T1 images, two experienced radiologists (authors CZ and LZ), each with over 10 years of experience, manually identified and segmented each stroke lesion, incorporating DWI, T2-weighted, and T2- FLAIR images for comprehensive lesion delineation. Additional imaging modalities, such as CT scans when available, were also used to enhance segmentation accuracy. These manually segmented lesions underwent further verification by two additional raters (authors JP and LG) to ensure consistency and accuracy.

**Figure 1:**
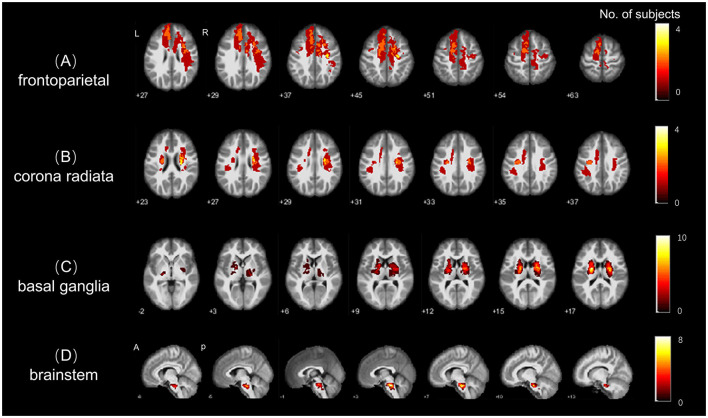
Stroke lesion topography. Lesion overlap for the four subgroups of stroke patients; with **(A–D)** indicating the spatial distributions of lesion in the **(A)** frontal-parietal, **(B)** radiation coronal, **(C)** basal ganglia, and **(D)** brainstem stroke subgroups, respectively. The colorbar represents the number of patients with stroke lesions in each region (voxel).

Subsequently, the segmented lesions were normalized into the MNI152 standard template () space using Statistical Parameter Mapping (SPM12, https://www.fil.ion.ucl.ac.uk) and the Clinical Toolbox (https://www.nitrc.org/projects/clinicaltbx/) (26), employing the lesion segment and normalization algorithm. The normalized segmentations were then resampled to a voxel size of 1 × 1 × 1 mm3 to facilitate lesion overlay (Figure 1) and creation of masks for preprocessing of R-fMRI data.

### 2.5 Preprocessing of R-fMRI data

R-fMRI data were preprocessed using conventional methods with the Data Processing and Analysis for Brain Image (DPABI, https://rfmri.org/DPABI) software (27), Statistical Parametric Mapping (SPM12, http://www.fil.ion.ucl.ac.uk), and MATLAB (https://www.mathworks.com). Briefly, R-fMRI data underwent (1) removal of the first 10 functional volumes, (2) slice timing correction, (3) realignment, estimation and correction for head motion (Friston 24) (28), and (4) co-registration with individual T1 anatomical images. The T1 images were segmented into gray matter (GM), white matter, and cerebrospinal fluid (CSF) classes, and spatially normalized into the MNI152 space using the non-linear diffeomorphic algorithm DARTEL (29). The co-registered functional volumes were then warped into the MNI152 space using deformative information from individual T1 to MNI template, and spatially smoothed using a Gaussian blur with a full width half maximum (FWHM) of 6 mm.

The spatially smoothed functional volumes were then regressed out linear trends, Friston 24 head motion parameters (28), mean frame-wise displacement (FD) (30, 31), average signals from the white matter and CSF classes. The regressed functional volumes were then temporally filtered within the frequency range of < 0.1 Hz, and finally co-registered to the mean symmetrical brain template generated from the DARTEL-processed T1 images.

### 2.6 Homotopic functional connectivity

Homotopic FC was computed on the symmetrical functional volumes using two complementary methods: (1) Voxel-wise mirrored homotopic connectivity (VMHC) (11). VMHC was obtained by calculating the *Pearson's* correlation coefficient, *r*, of the filtered resting-state BOLD signals between interhemispheric homotopic voxels, generating symmetrical homotopic functional connectivity (VMHC) map between the left and right hemispheres. (2) ROI-wise functional homotopy. As a validation, we also registered the symmetrical functional volumes to a calibrated and widely used homotopic atlas, Atlas of Intrinsic Connectivity of Homotopic Areas (AICHA) (32), which parcellates the hemisphere into 192 pairs of homotopic brain regions, allowing for analysis at the region level and enhancing the interpretation of brain functions. By calculating the functional connectivity (*r*) of the 192 pairs of homunculus brain regions, ROI-level homotopic connectivity can be obtained. These final voxel- and ROI-wise homunculus connections were *r* to *Z* transformed to enhance normality and facilitate group comparisons.

### 2.7 Quality assurance

To mitigate the influence of stroke lesions on preprocessing, lesion masks were applied during the preprocessing of both functional and structural images. This approach conforms the calculation to non-lesion brain tissues, thereby minimizing the lesions' impact on the results.

### 2.8 DTI data processing

DTI data was preprocessed using the Pipeline for Analyzing Brain Diffusion Images (PANDA, http://www.nitrc.org/projects/panda) (33), the FMRIB's Software Library (FSL ver 6.0.4, https://fsl.fmrib.ox.ac.uk/fsl/fslwiki), and MATLAB within a Linux environment. The preprocessing steps included quality inspection of imaging data, brain tissue extraction, head motion and eddy current correction. Each diffusion-weighted image was co-registered with the b0 image using an affine transformation. Diffusion tensors and metrics, such as fractional anisotropy (FA), were then calculated. The derived metrics were then non-linearly registered into the MNI152 standard space and resampled into 1 mm^3^ voxels. Spatial smoothing was applied using Gaussian kernels with a 6 mm full width at half maximum (FWHM). Spatially smoothing was applied using Gaussian kernels with a 6 mm full width half maximum (FWHM).

### 2.9 Brain behavior relationship

To investigate the association between patient motor function scores and alterations in VMHC, we conducted *Pearson's* linear correlation analyses within clusters that exhibited significant between-group differences. Specifically, for each subgroup, we extracted the mean Z-transformed VMHC within clusters that showed significant differences between groups. To adjust for multiple comparisons, we applied the *Bonferroni* correction, ensuring a stringent control over potential false positives (34). All correlation analyses were subjected to Bonferroni correction, setting a significance threshold at *p* < 0.05.

### 2.10 Statistical analysis

For voxel-wise imaging measures, a one-way analysis of variance (ANOVA) was used to assess differences in VMHC across the five groups, with age, gender, and education levels accounted for as nuisance variables. Following this, *post-hoc* analysis was conducted using two-sample independent *t*-tests to compare each pair of groups within the regions that showed significant differences in the ANOVA. Adjustments for multiple comparisons were made using the voxel-level family-wise error rate (FWE) technique (35), with a significance threshold set at *p* < 0.05 and a minimum cluster size of ≥20 voxels. For demographic, neurobehavioral and motor variables, SPSS (version 16.0; Chicago, IL) was used to examine normality, compare clinical, neurobehavioral and demographical data, and conduct correlation analysis. The *Shapiro-Wilk* test was used to assess normality. Clinical measure group comparisons were performed using one-way ANOVA or two-sample *t*-tests for continuous variables, and Chi-square test for categorical variables.

## 3 Results

### 3.1 Demographics and clinical data

Table 1 summarizes the demographics, clinical, and neurobehavior data for all participants. The patient subgroups and HC had comparable demographics (gender, age, and education years) and underlying conditions (hypertension, smoking, and alcohol drinking; *ps* > 0.05). However, there were significant between-group differences in motor and cognitive ratings for patient subgroups and HC, notably in NIHSS (*F* = 11.755, *p* < 0.001), the FMA (*F* = 12.504, *p* < 0.001), the MMSE (*F* = 2.971, *p* = 0.024), and the MBI (*F* = 11.110, *p* < 0.001).

### 3.2 Voxel-wise homotopy

#### 3.2.1 Frontoparietal stroke

**Figure 2:**
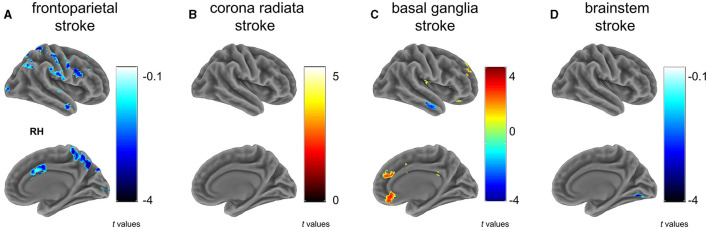
Between group differences on VMHC. Subfigures **(A–D)** show voxel-wise VMHC differences between each stroke subgroup vs. matched healthy controls. These results were corrected at a voxel-wise *p* < 0.001 and a cluster-wise *p* < 0.05, which corresponds to a cluster-wise *p* < 0.05 with FWE correction. The colorbar represents *t*-statistics for two-sample independent *t*-tests, with cold colors indicating the patients had lower VMHC than controls and hot colors showing the patients had higher VMHC than controls. RH, right hemisphere. **(A)** frontoparietal stroke, **(B)** corona radiata stroke, **(C)** basal ganglia stroke, and **(D)** brainstem stroke.

The frontoparietal group (G1) showed a significant reduction in VMHC across various regions compared to controls (Figure 2A and Table 2), particularly in clusters including somatosensory/motor, lateral parietal lobule, superior lateral temporal, opercular part of inferior frontal gyrus, supramarginal gyrus, anterior cingulate cortex, cerebellum_crus1, and cerebellum_crus2 (Figure 2A and Table 2).

**Table 2:** Results of VMHC among groups and the *post-hoc* multiple comparison test.

**Item**	**Peak region**	**MNI coordinates**			**Size (voxels)**	***t*-value**
		***x***	***y***	***Z***		
G1 > HC	Precuneus	3	−75	45	347	−4.737
	Parietal_Sup	15	−48	63	347	−4.573
	Temporal_Inf	57	−6	−36	70	−4.052
	Frontal_Mid_2	45	33	33	47	−4.040
	SupraMarginal	60	−30	24	140	−3.996
	Temporal_Sup	69	−12	12	140	−3.564
	Frontal_Inf_Oper	36	6	27	115	−3.909
	Cingulate_Mid	3	9	36	94	−3.795
	ACC_Sup	3	24	21	94	−3.191
	Frontal_Sup_Medial	9	33	57	54	−3.627
	Occipital_Mid	30	−99	0	58	−3.491
	Calcarine	12	−90	3	58	−2.922
	Cerebellum_Crus1	24	−84	−33	44	−3.420
	Cerebellum_Crus2	39	−72	−48	44	−2.907
	Occipital_Sup	33	−69	42	73	−3.355
	Precentral	57	0	39	156	−3.304
	Parietal_Inf	51	−36	54	78	−3.072
G2 > HC	ParaHippocampal	15	−6	−21	123	5.239
	Cerebellum_9	6	−63	−48	159	2.803
G3 > HC	Frontal_Sup_2	21	39	45	80	4.375
	Frontal_Med_Orb	9	33	−12	105	3.582
	Temporal_Inf	57	−3	−36	65	−3.743
	Cerebellum_9	12	−48	−54	70	4.779
	Cerebellum_8	33	−39	−48	47	4.171
G4 > HC	Lingual	15	−69	−9	56	−4.195

Regions were automatically labeled using the AAL3 atlas. x, y, and z = Montreal Neurological Institute (MNI) coordinates in the left-right, anterior-posterior, and inferior-superior dimensions, respectively. G1, frontal-parietal; G2, radiation coronal; G3, basal ganglia; and G4, brainstem stroke subgroups, respectively.

Sup, Superior; Inf, Inferior; Mid, Middle; Orb, Orbital; Oper, opercular; ACC, anterior cingulate cortex.

These results were reported with BSPMVIEW (http://www.bobspunt.com/software/bspmview/).

#### 3.2.2 Radiation coronal stroke

In contrast to the HC, patients with radiation coronal stroke (G2) exhibited significantly higher VMHC in the parahippocampal (*t* = 5.239, *p* < 0.05) and cerebellum_9 regions (*t* = 2.803, *p* < 0.05; Figure 2B and Table 2).

#### 3.2.3 Basal ganglia stroke

Similarly, in contrast to the HC, the basal ganglia group exhibited both significantly lower VMHC in the inferior temporal gyrus (*t* = −3.743, *p* < 0.05), and higher VMHC in the superior frontal gyrus (*t* = 4.375, *p* < 0.05), orbital portion of the superior frontal gyrus (*t* = 3.582, *p* < 0.05), cerebellum_8 (*t* = 4.171, *p* < 0.05) and cerebellum_9 (*t* = 4.779, *p* < 0.05; Figure 2C and Table 2).

#### 3.2.4 Brain stem stroke

The brain stem group exhibited significantly lower VMHC in the lingual and fusiform regions (*t* = −4.195, *p* < 0.05; Figure 2D and Table 2).

### 3.3 Atlas- and tract-based comparisons on white matter integrity

**Figure 3:**
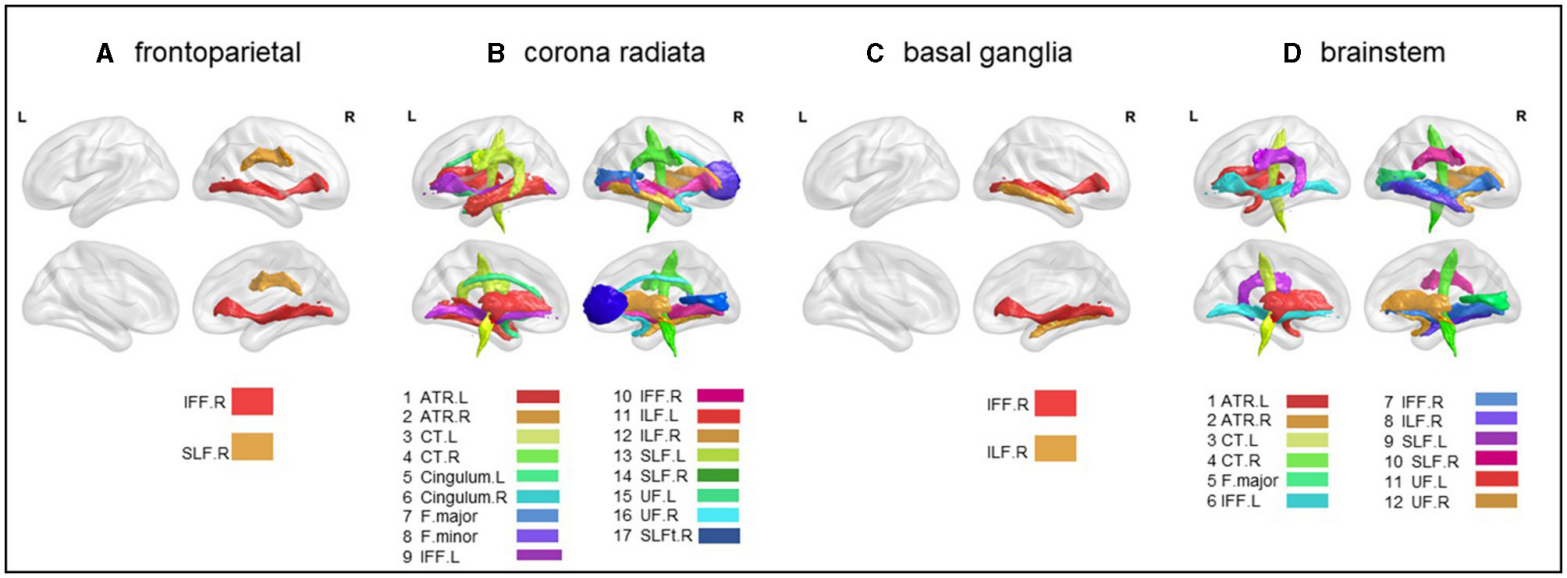
Atlas-based comparison on white matter integrity. Subfigures **(A–D)** show major white matter fiber tracts with reduced FA in different stroke patient subgroups. These results were corrected for *Bonferroni*, i.e., at a *p-*value < 0.05/20 = 0.0025. L, left; R, right; IFF, inferior fronto-occipital fasciculus; SLF, superior longitudinal fasciculus; ATR, anterior thalamic radiation; CT, corticospinal tract; F.major, forceps major; F.minor, forceps minor; ILF, inferior longitudinal fasciculus; UF, uncinate fasciculus. **(A)** frontoparietal, **(B)** corona radiata, **(C)** basal ganglia, and **(D)** brainstem.

To understand how subgroups of stroke patients involve different white matter tracts, leading to potentially reduced white matter integrity, we used the Johns Hopkins University (JHU) white-matter tractography atlas to perform both atlas-level (50 white matter parcellations) and tract-level (20 main tracts) analyses. Based on these atlases, we reported white matter integrity involvement implicated in different stroke subgroups (Figure 3 and Table 3).

**Table 3:** Results showing atlas- and tract-based comparisons on white matter integrity.

**Group**	**Item**	***t*-value**	***p*-value**	**Mean FA difference**	**Std. error difference**
G1 > HC	IFF.R	−2.110	0.044	−0.017	0.008
	SLF.R	−2.247	0.033	−0.016	0.007
G2 > HC	ATR.L	−3.419	0.002	−0.037	0.011
	ATR.R	−4.231	0.000	−0.041	0.009
	CT.L	−2.133	0.041	−0.021	0.009
	CT.R	−3.630	0.001	−0.037	0.010
	Cingulum.L	−3.135	0.004	−0.041	0.013
	Cingulum.R	−2.545	0.016	−0.039	0.015
	F.major	−2.835	0.008	−0.040	0.014
	F.minor	−2.683	0.012	−0.033	0.012
	IFF.L	−3.740	0.001	−0.032	0.008
	IFF.R	−5.065	0.000	−0.044	0.009
	ILF.L	−3.854	0.001	−0.031	0.008
	ILF.R	−4.324	0.000	−0.039	0.009
	SLF.L	−4.008	0.000	−0.027	0.007
	SLF.R	−5.103	0.000	−0.032	0.006
	UF.L	−4.272	0.000	−0.045	0.010
	UF.R	−3.433	0.002	−0.036	0.010
	SLFt.R	−2.881	0.007	−0.045	0.016
G3 > HC	IFF.R	−2.481	0.016	−0.016	0.007
	ILF.R	−2.078	0.042	−0.014	0.007
G4 > HC	ATR.L	−2.459	0.018	−0.018	0.007
	ATR.R	−2.305	0.026	−0.020	0.009
	CT.L	−2.676	0.010	−0.023	0.008
	CT.R	−2.101	0.041	−0.017	0.008
	F.major	−2.217	0.032	−0.026	0.012
	IFF.L	−2.343	0.024	−0.015	0.007
	IFF.R	−2.816	0.007	−0.022	0.008
	ILF.R	−3.016	0.004	−0.023	0.008
	SLF.L	−2.312	0.025	−0.015	0.007
	SLF.R	−2.344	0.023	−0.018	0.007
	UF.L	−2.343	0.024	−0.017	0.007
	UF.R	−2.490	0.017	−0.022	0.009

G1, frontal-parietal; G2, radiation coronal; G3, basal ganglia; and G4, brainstem stroke subgroups, respectively.

L, left; R, right; IFF, inferior fronto-occipital fasciculus; SLF, superior longitudinal fasciculus; ATR, anterior thalamic radiation; CT, corticospinal tract; F.major, forceps major; F.minor, forceps minor; ILF, inferior longitudinal fasciculus; UF, uncinate fasciculus.

#### 3.3.1 Frontoparietal subgroup

Significant lower FA in the right inferior fronto-occipital fasciculus (*t* = −2.110, *p* < 0.05) and the right superior longitudinal fasciculus (*t* = −2.247, *p* < 0.05) were observed (Figure 3A and Table 3).

#### 3.3.2 Radiation coronal subgroup

As compared to the HC, the radiation coronal subgroup showed significantly lower FA in various white matter tracts, including the bilateral anterior thalamic radiation (left, *t* = −3.419, *p* < 0.05; right, *t* = −4.231, *p* < 0.05), bilateral corticospinal tract (left, *t* = −2.133, *p* < 0.05; right, *t* = −3.630, *p* < 0.05), bilateral cingulum (left, *t* = −3.135, *p* < 0.05; right, *t* = −2.545, *p* < 0.05), bilateral inferior fronto-occipital fasciculus (left, *t* = −3.740, *p* < 0.05; right, *t* = −5.065, *p* < 0.05), bilateral inferior longitudinal fasciculus (left, *t* = −3.854, *p* < 0.05; right, *t* = −4.324, *p* < 0.05), bilateral superior longitudinal fasciculus (left, *t* = −4.008, *p* < 0.05; right, *t* = −5.103, *p* < 0.05), bilateral uncinate fasciculus (left, *t* = −4.272, *p* < 0.05; right, *t* = −3.433, *p* < 0.05), right superior longitudinal fasciculus (temporal; *t* = −2.881, *p* < 0.05), and the forceps major (*t* = −2.835, *p* < 0.05) and forceps minor (*t* = −2.683, *p* < 0.05; Figure 3B and Table 3).

#### 3.3.3 Basal ganglia subgroup

As compared to the HC, the basal ganglia subgroup exhibited significantly lower FA in the right inferior fronto-occipital fasciculus (*t* = −2.481, *p* < 0.05) and the right inferior longitudinal fasciculus (*t* = −2.078, *p* < 0.05; Figure 3C and Table 3).

#### 3.3.4 Brain stem subgroup

As compared to the HC, the brain stem subgroup showed significantly lower FA in the bilateral anterior thalamic radiation (left, *t* = −2.459, *p* < 0.05; right, *t* = −2.305, *p* < 0.05), bilateral corticospinal tract (left, *t* = −2.676, *p* < 0.05; right, *t* = −2.101, *p* < 0.05), bilateral inferior fronto-occipital fasciculus (left, *t* = −2.343, *p* < 0.05; right, *t* = −2.816, *p* < 0.05), bilateral superior longitudinal fasciculus (left, *t* = −2.312, *p* < 0.05; right, *t* = −2.344, *p* < 0.05), bilateral uncinate fasciculus (left, *t* = −2.343, *p* < 0.05; right, *t* = −2.490, *p* < 0.05), forceps major (*t* = −2.217, *p* < 0.05), and the right inferior longitudinal fasciculus (*t* = −3.016, *p* < 0.05; Figure 3D and Table 3).

### 3.4 Association analysis

**Figure 4:**
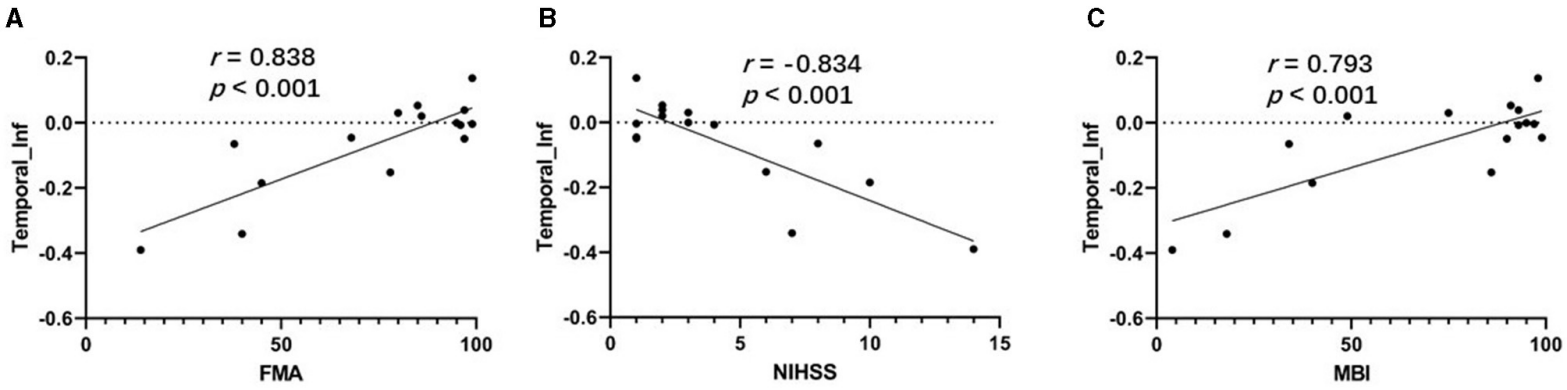
**(A)** Correlation between z-values of inferior temporal gyrus in VMHC in G1 and FMA. **(B)** Correlation between z-values of inferior temporal gyrus in VMHC in G1 and NIHSS. **(C)** Correlation between *z*-values of inferior temporal gyrus in VMHC in G1 and MBI. Temporal_Inf, inferior temporal gyrus; FMA, Fugl-Meyer Assessment scale; NIHSS, National Institutes of Health Stroke Scale; MBI, Modified Barthel Index.

As shown in Figures 4A, C, the correlation analysis revealed positive associations between the FMA and MBI scores and the z-values of the inferior temporal gyrus in VMHC in G1 (*r* = 0.838, *p* < 0.001, and *r* = 0.793, *p* < 0.001, respectively). Figure 4B show a negative correlation (*r* = −0.834, *p* < 0.001) between the NIHSS score and the z-values of the inferior temporal gyrus in VMHC in G1. Regarding cognitive evaluations, there was no statistically significant correlation found between the MMSE score (*r* = 0.451, *p* = 0.091) and the *z*-values of the inferior temporal gyrus in VMHC in G1 (Table 4). In G2, G3, and G4, no statistically significant association analysis was observed.

**Table 4:** The correlation results between brain region and clinical measures.

**Item**	**Region**	**NIHSS**	**FMA**	**MMSE**	**MBI**
VMHC	Temporal_Inf	*r* = −0.834^**^	*r* = 0.838^**^	*r* = 0.451	*r* = 0.793^**^
G1		*p* < 0.001	*p* < 0.001	*p* = 0.091	*p* < 0.001

^**^p < 0.01.

VMHC, Voxel-mirrored homotopic connectivity; G1, frontoparietal group; Temporal_Inf, inferior temporal gyrus; NIHSS, National Institutes of Health Stroke Scale; FMA, Fugl-Meyer Assessment scale; MMSE, Mini-Mental State Examination; MBI, Modified Barthel Index.

## 4 Discussion

In this study, we investigated how acute ischemic stroke in different locations affects interhemispheric homotopic functional and structural connectivity, as well as their associations with motor and cognitive scores. Our findings include: (1) Frontoparietal strokes were associated with reduced homotopic connectivity in the somatosensory motor cortex; (2) strokes in the corona radiata showed increased connectivity in the parahippocampal and cerebellar regions; (3) basal ganglia strokes exhibited both lower homotopic functional connectivity in the inferotemporal gyrus and higher homotopic functional connectivity between the ventrolateral prefrontal cortex and the cerebellum; (4) brainstem strokes were linked to decreased homotopic connectivity in higher visual processing areas. These distinct lesion locations led to diverse changes in interhemispheric homotopic connectivity, providing novel evidence for the mapping of brain connectivity damage.

Consistent with some of our hypotheses and previous reports (36, 37), strokes in different locations resulted in alterations in homotopic connectivity not solely within motor circuits, despite all presenting with early post-stroke motor functional impairments. Studies on the normal variations in the healthy human brain have indicated that interhemispheric homotopic connectivity exhibits regional variability, with the strongest interhemispheric connections observed in primary cortices such as somatosensory, auditory, and visual areas, and relatively weaker or more individually variable connections in associative cortices like the frontoparietal network and the lateral temporal lobe (11, 38). These earlier findings align with recent years' recognition and discovery of gradients in the human brain (39). In our study, frontoparietal strokes displayed pronounced reductions in widespread homotopic connectivity, including within networks supporting motor functions. This change corroborates previous reports (16) and may also reflect the dense, extensive involvement of network hub nodes in this region, leading to significant alterations in interhemispheric neurodynamics.

In contrast, strokes in the corona radiata did not exhibit pronounced reductions in homotopic connectivity within the cortical somatosensory/motor network areas. Instead, they showed enhanced homotopic connectivity in the parahippocampal and cerebellar regions. Although this outcome is contrary to our predictions, it is interpretable. The corona radiata contains a series of projection fibers that connect the cortex and the brainstem through the internal capsule. Damage in this area may lead to abnormal communication between cortical and subcortical areas, resulting in an imbalance of cortical excitation and inhibition, which could manifest as compensatory reductions and increases in homotopic connectivity. Another possibility is that the observed increase in homotopic connectivity in cerebellar regions reflects a neural mechanism for motor functional impairment. This result is largely in line with previous reports (40). A prior study on ischemic stroke damage in the corona radiata and pons also reported both increases and decreases in local spontaneous activity (such as local coherence ReHo, voxel-level degree centrality), interpreting these changes as more likely reflecting anatomical specificity rather than pathway specificity (40).

Our results indicate that basal ganglia strokes primarily showed changes in interhemispheric connectivity within networks such as the default mode network (including the ventromedial prefrontal cortex, posterior cingulate cortex, and lateral temporal lobe), the salience/cognitive control network (dorsal anterior cingulate cortex and pre-supplementary motor area), and parts of the somatosensory motor network. These changes, mostly characterized by enhanced interhemispheric homotopic functional connectivity, are not in line with previous studies (41). The default mode network, implicated in social cognition, abstract thinking, and autobiographical memory, is known to be vulnerable in neurological disorders including stroke and traumatic brain injury (42, 43). Anatomically, the ventromedial prefrontal cortex is associated with decision-making and self-referential processes (44), while the posterior cingulate cortex plays a role in autobiographical memory, memory encoding, and consolidation, which may relate to cognitive impairments observed in these patients (45). The dorsal anterior cingulate cortex and pre-supplementary motor area, central to the salience network, are involved in balancing interoceptive awareness and external attention, serving as a primary network basis for pathologies including mild cognitive impairment following traumatic brain injury (46) and autism (47), aligning with our recent findings on cognitive impairment post-hemorrhagic stroke in the basal ganglia (48). Furthermore, we identified changes in the homotopic functional connectivity of some somatosensory motor areas, consistent with our predictions.

Brainstem strokes showed reduced interhemispheric homotopic functional connectivity in higher visual cortices, with fewer changes in interhemispheric homotopic functional connectivity in other motor-related cortical areas. Similar to the results of corona radiata strokes, these changes might relate to projection pathways, with relatively less impact on cortical interhemispheric integration.

Following the discussion on how different ischemic stroke locations affect interhemispheric homotopic functional connectivity, we further analyzed changes in white matter structural connectivity. Overall, subgroups with prominent changes in homotopic functional connectivity, such as those with frontoparietal and basal ganglia strokes, mainly showed reduced white matter integrity of association fibers (lower FA values). Conversely, subgroups with less change in homotopic functional connectivity, like those with corona radiata and brainstem strokes, exhibited more pronounced reductions in the white matter integrity of projection fibers. This mismatch between function and structure may reflect different coupling mechanisms that future research needs to explore further. This inconsistency may also indicate different neural mechanisms underlying post-stroke motor impairments, necessitating varied diagnostic and treatment strategies. Indeed, the recovery from post-stroke motor impairments shows significant individual variability, ranging from days to months or years after a stroke, and even leading to permanent motor disability.

### 4.1 Limitations and future directions

There are some limitations of this study that must be addressed. First, although this study focused on poststroke movement disorders, there are large differences in the number of participants in each subgroup. Yet, the amount of fiber regions used for the analysis is pretty small (50 brain regions and 20 major tracts), all of which limit the quality of statistical analysis and interpretability of findings. The grouping resulted in a small subgroup sample size, and future studies need to delineate finer lesion subgroups based on larger samples. Second, since the patients included in this study were in the acute phase of stroke, the differences in drug application, potential brain tissue edema, head movements during scanning, and pre-morbid underlying conditions resulted in unknown effects on brain function, which may need to be validated by further animal experiments based on controlled conditions. Third, because we used group-level lesion masks in the preprocessing, VMHC involving within the lesions was not considered, leading to a bit difficult interpretation of the results. Fourth, there were significant between-group differences in motor and cognitive scores among patient subgroups and HCs. These differences present difficulties in the extent to which differences in measured motor and cognitive scores explain differences in VMHC, and we will seek ways to resolve this issue in subsequent studies. We will validate these findings in larger cohort studies in the future and measure the extent to which VMHC alterations may predict motor recovery via longitudinal follow-ups.

## 5 Conclusion

Our results suggest that post-stroke motor deficits in different regions implicate different links from cortical to subcortical areas. Alterations in lesion topography and regional functional homotopy provide new insights into the understanding of neural basis of post-stroke dyskinesia and also inform potential individualized precisive targets.

## Data availability statement

The original contributions presented in the study are included in the article/supplementary material, further inquiries can be directed to the corresponding authors.

## Ethics statement

The studies involving humans were approved by the Ethics Committee of the First College of Clinical Medical Science of China Three Gorges University. The studies were conducted in accordance with the local legislation and institutional requirements. The participants provided their written informed consent to participate in this study.

## Author contributions

CZhao: Formal analysis, Writing – original draft. CZhan: Writing – original draft, Formal analysis. LZ: Data curation, Writing – original draft. LC: Data curation, Writing – original draft. XX: Data curation, Writing – original draft. JP: Data curation, Writing – original draft. JC: Data curation, Writing – original draft. LG: Formal analysis, Writing – original draft. CY: Conceptualization, Writing – review & editing, Supervision. HX: Conceptualization, Supervision, Writing – review & editing.
